# P-565. Retrospective Analysis of Antimicrobial Drug Shortages in Japan from 2021 to 2024

**DOI:** 10.1093/ofid/ofaf695.780

**Published:** 2026-01-11

**Authors:** Hilary Osaka, Yasuaki Tagashira, Keito Imada, Koh Okamoto, Akane Takamatsu, Noritaka Sekiya, Yoshiaki Gu

**Affiliations:** Institute of Science Tokyo, Bunkyo-ku, Tokyo, Japan; Institute of Science Tokyo, Bunkyo-ku, Tokyo, Japan; Institute of Science Tokyo, Bunkyo-ku, Tokyo, Japan; Institute of Science Tokyo, Bunkyo-ku, Tokyo, Japan; Institute of Science Tokyo, Bunkyo-ku, Tokyo, Japan; Institute of Science Tokyo, Bunkyo-ku, Tokyo, Japan; Institute of Science Tokyo, Bunkyo-ku, Tokyo, Japan

## Abstract

**Background:**

Limited access to safe and effective antimicrobials is a global concern that adversely affects patient outcomes and public health, especially amid the growing threat of antimicrobial resistance. Identifying the underlying causes of the shortages is vital, as no single solution can address all cases. Herein, we comprehensively evaluated nationwide antimicrobial drug shortages in Japan.

**Figure:**
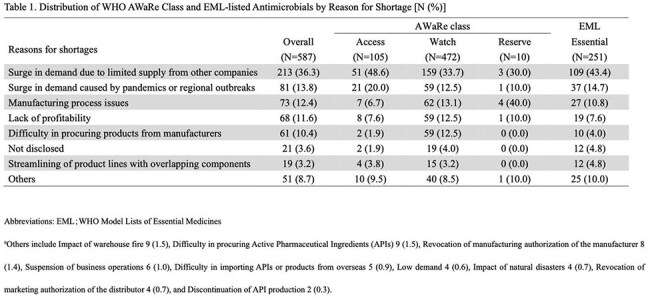

**Methods:**

We conducted a retrospective study using shortage notifications submitted by pharmaceutical companies in Japan from April 2021 to March 2024. We collected shortage data from DrugShortage.jp, a database tracking pharmaceutical supply status. Missing data were supplemented by reviewing company websites and contacting companies. Antimicrobials were categorized by antimicrobial class, WHO AWaRe classification, and the WHO Model List of Essential Medicines (EML). We also analyzed the reported causes, duration, and resolution status of each shortage.

**Results:**

A total of 587 antimicrobial shortage notifications were identified during the study period. The most commonly affected antimicrobial classes were cephalosporins, with 200 events (34.1%), followed by quinolones, macrolides and penicillins with 102 (17.4%), 83 (14.1%) and 80 (13.6%) events respectively. By WHO AWaRe classification, 105 (17.9%) were for Access medications, 472 (80.4%) as Watch and 10 (1.7%) as Reserve, and 251 (42.8%) were for those listed on the EML. The most reported cause of shortage was increased demand due to limited supply from other companies, followed by demand surges related to pandemics or regional outbreaks, and manufacturing process issues (Table 1). The median shortage duration was 285 days (range: 9-891 days). At the end of the study period, 116 (19.8%) were resolved, 250 (42.6%) remained ongoing, and 221 (37.6%) were withdrawn from production or sales.

**Conclusion:**

This study revealed that antimicrobial shortages in Japan affect a wide range of antimicrobials including essential ones and are often prolonged. Shortages frequently result from disruptions in supply from other manufacturers, though other factors were also involved. Our findings emphasize the need for multifaceted policy responses to ensure a stable and resilient antimicrobial supply system.

**Disclosures:**

Koh Okamoto, MD, MS, PhD, Becton, Dickinson and Company: Honoraria|Shionogi: Honoraria|Terumo: Honoraria|Thermo Fisher Scientific: Honoraria

